# A Model Predictive Control for Lot Sizing and Scheduling Optimization in the Process Industry under Bidirectional Uncertainty of Production Ability and Market Demand

**DOI:** 10.1155/2022/2676545

**Published:** 2022-09-30

**Authors:** Qi Tang, Yiran Wang

**Affiliations:** School of Management, Shenyang University of Technology, Shenyang 110870, China

## Abstract

In the face of bidirectional uncertainty of market demand and production ability, this paper establishes a multiobjective mathematical model for lot sizing and scheduling integrated optimization of the process industry considering both material network and production manufacturing and finds the optimal decision of the model through model predictive control to minimize total completion time and total production cost. While realizing the model predictive control proposed in this paper, the Elman neural network predicts the relevant parameters required by learning historical orders for the uncertain market demand and equipment production ability. Then, the calculation formulas of product supply and demand matching and equipment production ability are formed and introduced into the next stage of the model as a constraint condition. In addition to the above constraints for constructing lot sizing and scheduling integrated models in the process industry, this paper also considers both the material network and production manufacturing and uses the IMOPSO algorithm to solve the problem iteratively. So far, a complete model predictive control can be generated. Through the model predictive control, the production system can respond in advance, make appropriate changes to offset the foreseeable interference, and obtain the lot sizing and scheduling scheme considering bidirectional uncertainty, thereby improving the system's overall robustness. Finally, this paper realizes the model's predictive control process through example simulation and analyzes the operation results combined with the scheduling Gantt chart to verify the applicability and effectiveness of the model.

## 1. Introduction

With the vigorous development of the industrial Internet, the market demand has gradually become dynamic and diversified. As a result, in the context of the era of big data, the traditional manufacturing industry must make new changes in line with the market to gain a head start in the new wave of the times [1, 2]. As a leader in the manufacturing industry, it is in this transformation that the process industry has shifted its focus from the original quality-centered to the customer's individualized demand. The production mode has changed from the original small variety and large lot to the current multivariety and small lot [3, 4]. Among them, the transformation centered on customer needs requires enterprises to grasp the external market's needs accurately. However, the actual market is full of various uncertainties. Consumers' demand preferences for products significantly affect the production planning of manufacturing companies. It can be seen that the existing historical order demand is analyzed to deal with the uncertain future market is a vital link [5, 6]. In addition, with the growth of the processing cycle, a series of uncertain situations such as wear, aging, and even equipment failure will occur in the actual application process [7, 8]. The abovementioned conditions will lead to a decline in its production ability. The production ability is also full of uncertainty. In order to make the existing theoretical results more suitable for the actual situation, based on this production background, this paper focuses on how the process industry responds to the uncertain external environment considering the bidirectional uncertainty of market demand and production ability and formulates a formula for MES.

In the existing research on lot sizing and scheduling in the process industry, there are many pieces of literature that are closely related to market demand and equipment production ability. For example, based on the production background of automobile manufacturing enterprises, Hakeem-Ur-Rehman et al. proposed a mathematical model that the production schedule can change with the change in demand, which can maximize the production quantity and the sequence of different production stages while determining the production quantity and sequence to reduce the total cost of production and inventory [9]. Bergamini et al. optimized production schedules for home appliance manufacturers to meet planned market demands while minimizing processing time and the number of split lots [10]. Nobil et al. established a mathematical model to minimize the total inventory cost after considering the maximum order demand, equipment production ability, and budget constraints so that the optimal cycle length of the product and the number of delivery lots can be determined. [11]. When Georgiadis et al. studied lot sizing and scheduling production in large dairy plants in Greece, she fully considered inventory constraints, material balances, and equipment production ability, rationalizing the decision-making process for production scheduling [12]. From the abovementioned examples, it is not difficult to find whether it is market demand or equipment production ability, and the existing research mostly starts from the determined value to realize the lot sizing and scheduling of the process industry. However, with the rapid development of the economy, the diversification of the market has led to more uncertainty in consumer demand. In the production process, a series of uncertain situations such as wear and aging of equipment will also cause its production ability to remain unfixed. Whether it is uncertain market demand or equipment production ability, there is still much room for exploration in the process industry's current research field of lot sizing and scheduling. Therefore, to make the current production problems more realistic, this paper takes the uncertain market demand and equipment production ability into consideration for the first time and applies them to the process industry's lot sizing and scheduling problems.

As the core of control theory, the control method has always been one of the directions of great interest to scholars. Generally speaking, standard control methods can be divided into optimal control, fuzzy control, model predictive control, and neural network control methods. Among them, optimal control refers to a control method that makes the determining system performance index reach the maximum value (or minimum value) under the given constraint conditions. For example, when implementing optimal control, Zamfirache et al. proposed a control method based on reinforcement learning that combines DQL and meta-heuristic GSA to initialize the weights and sums of NNs involved in learning and optimal control—aiming to be able to achieve the optimal reference tracking control objective autonomously [13]. Ahmed et al. used optimal control to determine the optimal scale and optimal timing to apply impulse control and gave an optimal strategy to minimize the objective (cost) function [14]. Klančar et al. proposed a new trajectory planning algorithm to generate minimum time trajectories of vehicles. It considers the driving constraints of maximum velocity and acceleration and finds a computationally efficient and time-optimized solution for a given initial and final configuration, which can be applied in path planning applications [15]. It can be seen that the implementation of optimal control has certain limitations, that is, it not only requires the system to be stable but also requires the system to achieve optimal performance indicators.

The fuzzy control method is a computer control method based on fuzzy mathematics and is composed of a fuzzy set theory, a fuzzy language, and a fuzzy logic [16]. It belongs to a kind of nonlinear intelligent control, which can transform human thinking and fuzzy [17] language to realize the effective control of the controlled object that cannot establish an accurate model. For example, Pozna et al. combined PF and PSO to propose a hybrid meta-heuristic optimization algorithm, which was then applied to optimally adjust a proportional-integral fuzzy controller for position control of a series of integrated servo systems [18]. Tim Chen et al. used the optimization of fuzzy control to tune the model and control parameters of a nonlinear system to asymptotically stabilize the system [19]. Mohammadzadeh A et al. proposed a robust fuzzy control method for lateral path tracking of autonomous road vehicles (ARVs), which can be effectively applied to the path tracking task of ARVs under a wide range of operating conditions and external disturbances [20]. It can be seen from the abovementioned examples that the fuzzy control method can describe the system with language-based fuzzy variables instead of numerical values so that the controller does not need to establish a complete mathematical model for the controlled object. However, obtaining fuzzy rules and membership functions is entirely based on experience with certain drawbacks.

Model predictive control is a particular control method developed in industrial production. It can realize the precise target control according to the prediction model of the control object and is widely used in optimal control with constraints. For example, Balta et al. proposed a closed-loop model predictive control (MPC) framework for PTA discrete event systems enabling real-time model update-based decision-making [21]. Shen et al. proposed an EWH intelligent dispatch control system that utilizes data-driven disturbance prediction in robust model predictive control (MPC) to accomplish various demand management objectives [22]. Chen et al. developed an improved MPC scheme and used it with a hybrid energy storage system for optimal power dispatch in intelligent grid systems [23]. However, when model predictive control is applied to a robust nonlinear system, there will be a significant model mismatch, and the control effect cannot be achieved.

The learning control method based on a neural network mainly relies on the neuron model for optimization and application, which can accurately approximate the system without analyzing the system model to form a network structure that can have an excellent, intelligent control effect and has both self-learning and high-speed resolution effect and has become widely used intelligent control technology. For example, Peng et al. developed an adaptive neural controller with a dynamic learning framework for robotic manipulators interacting with unknown environments in dead actuator zones [24]. Shi et al. designed an adaptive neural controller with a new neural weight update law, which ensures that the RBF neural network can accurately identify unknown systems and enables the estimated neural weights to converge to their ideal values [25]. Combining the adaptive neural network with ADRC design techniques, Liu et al. proposed a new dual-channel composite controller scheme, which can guarantee the tracking of the desired signal within a small domain of the origin [26]. From this, it is not difficult to see that the learning control method based on a neural network is more flexible than other control methods and can solve more complex control problems of unknown nonlinear systems, but it cannot realize control problems with constraints.

Therefore, to further solve problems such as model mismatch and lack of constraints, model predictive control based on neural networks has begun to receive extensive attention from scholars. For example, Li et al. proposed a model predictive control method based on a radial basis function neural network for the path tracking problem of underactuated surface ships with input saturation, parameter uncertainty, and environmental disturbance [27]. For the position control of a single-link flexible joint (FJ) robot, Zhang et al. proposed a nonlinear model predictive control technology based on recurrent neural networks and differential evolution optimization [28]. Núñez et al. proposed a model predictive control scheme (NNMPC) based on a recurrent neural network to complete the real realization of the control of an industrial slurry thickener [29]. Compared with the general learning control method, the model predictive control based on a neural network has a unique optimization tool, which can make the system achieve stability and solve the decision plan with the best performance index. The system model in the model predictive control can deal with unknown information or nonlinear system problems through its learning mechanism and does not depend on the mathematical model of the controlled object.

Based on the abovementioned analysis, it can be found that no matter what the model predictive control method may be, it mainly focuses on path tracking, power system, and intelligent control, but it is rarely involved in the field of process industry production scheduling. Therefore, from the perspective of process production scheduling, this paper proposes for the first time a multiobjective lot sizing and scheduling integrated optimization model for multiproduct switching production in the process industry after comprehensively considering the bidirectional uncertainty of market demand and production ability. The neural network-based model predictive control method obtains the optimal decision to minimize the total completion time and switching cost. Among them, during the implementation of model predictive control, the neural network realizes the prediction of uncertain variables, and the heuristic algorithm can solve the constructed multiobjective optimization model.

Different from previous studies, the innovation of this paper is mainly reflected in the following aspects: (1) applying the neural network-based model predictive control method to the production scheduling direction of the process industry; (2) when designing the process industry lot sizing and scheduling integrated optimization model, it can comprehensively consider the bidirectional uncertainty of market demand and production ability; (3) in the process of process industry production, not only the intermediate inventory caused by the material flow in the material network is considered but also the lots in the manufacturing process are considered; and (4) the Elman neural network, the optimization model, and the IMOPSO algorithm are combined to complete the model predictive control so that the process industry can respond to uncertain situations in advance and can formulate a corresponding production scheduling scheme.

## 2. Problem Description

The current market needs *p* products, the enterprise goes through *t* production orders, and each product in the production process will go through *s* production stages. Among them, *P*={1,2, ..., *p*}, *T*={1,2, ..., *t*}, and *S*={1,2, ..., *s*}. It is assumed that the market demand of the product *i* ∈ *P* in each order is denoted as *d*_*it*_. In process-based production, the material will have a certain conversion rate in the input and output, so the production of products in each stage is not completely equal. Let *θ*_*ij*_ denote the conversion rate of the product *i* ∈ *P* at stage *j* ∈ *S*. Usually, equipment will process and produce a variety of products. In the actual production process, the production of the product *i* ∈ *P* at any stage *j* ∈ *S* will be greater than the single production lot of equipment at that stage, so the product must be produced in lots at any stage.

In previous studies, the variables faced by the process industry production are primarily determined. However, the rise of the Internet economy has led to the diversification and dynamization of customer demand, resulting in enterprises' uncertainty of market demand when arranging future production plans. In addition, there are also a series of uncertainties such as equipment aging and failure in the production equipment. Especially in an automatic production line system, the process control is entirely driven by field data. However, once equipment failure occurs, the production system will be suspended, which may cause safety accidents, and the workload of the subsequent production recovery is hefty, thereby seriously affecting the production progress.

In order to solve the dilemma of bidirectional uncertainty, this paper establishes a multiobjective mathematical model of lot sizing and scheduling integration based on the process industry. Then, the optimal decision of the model is found through the model predictive control to minimize the total completion time and the total production cost. The specific model predictive control process is shown in Section 3.4.3. The model predictive control proposed in this study can not only consider the bidirectional uncertainty of market demand and production ability that may occur in the future but also enable the system to respond in advance and make appropriate changes to offset the predictable interference before the uncertain disturbance occurs, thereby improving the safety and stability of the on-site production process.

## 3. A Model Predictive Control Based on Integrated Optimization of Lot Sizing and Scheduling in the Process Industry

This section mainly relies on two elements: the integrated model of lot sizing and scheduling in the process industry and the implementation of MPC to drive decision-making updates. In the integrated model of lot sizing and scheduling in the process industry, the focus is to establish a decision-making optimization model in the MES system that can realize the integration of lot sizing and scheduling. The model is detailed enough to capture all the essential variables in the system. As for the realization of MPC, the Elman neural network is introduced to continuously learn to predict the trend of uncertain variables in the future, and then the variables are substituted into the model to update the internal decision-making of the system.

### 3.1. Dynamic Characterization

**Figure 1:**
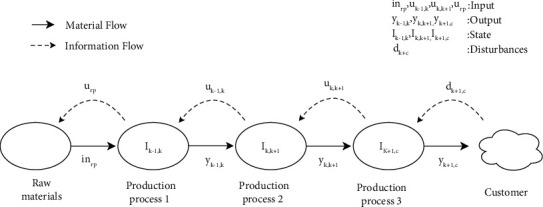
Mapping diagram of variables.

This paper's dynamic characterization in reference [30] defines the dynamic system's input, output, state, and disturbance.  Figure 1 shows the mapping between specific variables.

We can see from Figure 1 that the upstream output of each stage enters the production system as the input of the corresponding stage. The products (including semifinished products) produced in each process are connected to the next stage as the corresponding output of this stage. The status of the previous process in two adjacent processes is represented by the inventory between the two adjacent processes. Based on the fluctuation of customer demand for different products and the production ability of equipment changes with the processing time, the product demand and the production ability of the equipment appear as disturbances in the system. We thereby propose a dynamic model that considers all these variables with the abovementioned dynamic features in the next section. It should be noted that the system's input, output, and disturbance can be observed in real-time, while the state variables can be obtained by further calculation. The explanation of the symbols in all formulas in the article can seen in Table 1.

### 3.2. A Dynamic Optimization Model for Lot Sizing and Scheduling Integration in the Process Industry

#### 3.2.1. Nomenclature

#### 3.2.2. Problem Assumption

The assumptions that the lot sizing and scheduling integrated optimization model of the process industry studied in this paper are mainly divided into the following aspects:

1. Multiple products can be processed during processing, but all products pass through the same production stage in the sequence.
2. In the process of production, raw materials are continuously supplied.
3. Each piece of equipment can process multiple products, but the same equipment can only process one product simultaneously.
4. Each product can be divided into multiple lots, but each lot can only process one product simultaneously.
5. The product allows the material conversion rate during processing. That is, the input and output quantities of the process are allowed to be inconsistent.
6. Intermediate inventories are allowed between different processes in the production process, but the number of intermediate inventories is limited.
7. When the equipment on a particular process is switched from the production process of the current product to the production of the next product, the equipment needs to be cleaned and adjusted.
8. The demand for products and the production ability of equipment are uncertain.
9. This article assumes that an order cycle refers to the total processing time from the start of production to the complete end of the order. The cycle of each order is determined by the order quantity, not a fixed value.

Among them, assumptions 1-4 are the basic assumptions in the production process of the process industry, and assumptions 5-9 are the specific assumptions put forward by this paper on the process industry products based on the existing ones.

#### 3.2.3. Material Network

In the material network, the transmission of material flow is significant, which mainly depends on the input and output of each process and the intermediate inventory between two adjacent processes. The intermediate inventory is the state variable in the dynamic model established in this paper. Therefore, in this section, we discuss the logical relationship between each input variable and output variable and the calculation formula of the state variable.

First, because this article considers whether the materials of each lot of products can entirely convert between adjacent equipment in the actual situation, the input materials of a particular lot on the equipment should be the same on the adjacent equipment. The equipment of the stage produces the intermediate material after calculating the conversion rate, not the material input by the equipment of the adjacent previous stage. Therefore, the relationship between input variables and output variables can be expressed by equation (1):

(1) $$\begin{array}{c} \underset{k\in N}{\sum }y_{ijkt}=\underset{k\in N}{\sum }u_{ijkt}\theta_{ij},\forall i\in P,j\in S,t\in T. \end{array}$$

Due to its physical limitations, the equipment on the production line can only produce a limited number of products simultaneously. In order to rationalize the products' number in each lot, this paper limits the products' number in each order. The lot size should be within the processing ability of the equipment at the corresponding stage, and it can be expressed as shown in equation (2):

(2) $$\begin{array}{c} x_{ijkt}Bb_{ijt}^{\min}\leq u_{ijkt}\leq x_{ijkt}Bb_{ijt}^{\max^{\prime }}\forall i\in P,j\in S,k\in N,t\in T. \end{array}$$

It can be seen from the description in Section 3.1 that the state variable in the system corresponds to the intermediate inventory in the material network. This variable is usually unmeasurable, so it is necessary to calculate the actual state based on the currently measured input and output variables at each sampling time point. The specific equation is as follows:

(3) $$\begin{array}{c} I_{ijkk^{\prime }t}=I_{i,j-1,k^{\prime \prime }kt}+\underset{0\leq kk\leq k}{\sum }y_{ij,kk,t}-\underset{0\leq kk\leq k^{\prime }}{\sum }u_{i,j+1,kk,t},\forall i\in P,j\in S,j\neq jm,k\in N,k^{\prime }\in N,k^{\prime \prime }\in N,t\in T. \end{array}$$

Since the space in the production workshop is limited, there is also a quantitative limit range for the intermediate inventory. However, the intermediate inventory may not necessarily be within the reasonable range, so it needs to be constrained and restricted, as shown in equation (4):

(4) $$\begin{array}{c} 0\leq I_{ijkk^{\prime }t}\leq I_{ij}^{\max},\forall i\in P,j\in S,k\in N,k^{\prime }\in N,t\in T. \end{array}$$

#### 3.2.4. Production Manufacturing

In production manufacturing, the lot sizing of products on each device and the timing of the corresponding lots are critical. Each scheduling scheme must ensure no time conflict between lots and should minimize the amount of time during the production process–the number of switches.

First of all, in each order, each equipment can process multiple products, but when the equipment processes each product, each position can only have one lot production task at the most, which can be expressed by equation (5):

(5) $$\begin{array}{c} \underset{i\in P}{\sum }x_{ijkt}\leq 1,\forall j\in S,k\in N,t\in T. \end{array}$$

In addition, each position point does not necessarily have lot production tasks, that is, there may be virtual positions. If the position point is real, then $\underset{i\in P}{\sum }x_{ijkt}=1$, else $\underset{i\in P}{\sum }x_{ijkt}=0$. When the following position point is real, then $\underset{i\in P}{\sum }x_{ijkt}=\underset{i\in P}{\sum }x_{ij,k+1,t}$. Similarly, when the position point behind is virtual, then $\underset{i\in P}{\sum }x_{ijkt}>\underset{i\in P}{\sum }x_{ij,k+1,t}$. Therefore, the relationship between position points can be expressed by the following equation:

(6) $$\begin{array}{c} \underset{i\in P}{\sum }x_{ijkt}\geq \underset{i\in P}{\sum }x_{ij,k+1,t},\forall j\in S,k\in N,k\neq km,,t\in T. \end{array}$$

The order of lots is primarily involved in scheduling divided lots to be produced orderly. Because a single device on each production line can only produce one task simultaneously, there is a specific time relationship between the lot production tasks corresponding to each position point.

(7) $$\begin{array}{c} {\text{tstart}}_{ij,k+1,t}\geq {\text{tstart}}_{ij,kt}+q_{ij}x_{ijkt}+\lambda_{ij}u_{ijkt}\theta_{ij}\;x_{ijkt},\forall i\in P,j\in S,k\in N,k\neq km,t\in T. \end{array}$$

First the relationship between the adjacent two position points of the same product on the same machine in each order: if there are lot production tasks at the two adjacent position points on a particular machine and the same product is produced at the two position points, then the start time of the lot production task at the latter position point should not be less than the end time of the lot production task at the former position point, and this can be expressed as follows:

Second is the relationship between two adjacent position points of different products on the same machine in each order: if there are lot production tasks at two adjacent position points on a particular machine and different products are produced at two position points, then the start time of lot production tasks at the latter position point should not be less than the sum of the end time of lot production tasks corresponding to the former position point and the adjustment time between the two products, which can be expressed as follows:

(8) $$\begin{array}{c} tstart_{i'jkt}+q_{i'j}x_{i'jkt}+\lambda_{i'j}u_{i'jkt}\theta_{i'j}x_{ijkt}-Z\left(1-x_{i'jkt}\right)+T_{i'ijt}x_{ij,k+1,t}\leq tstart_{ij,k+1,t}\;,\forall i\in P,i^{\prime }\in P,j\in S,k\in N,t\in T\text{.} \end{array}$$

Third is the relationship between the same lot of the same product on the adjacent machine in each order: if the same lot of a product has many production tasks on the adjacent machine, then the start time of the lot production task on the back machine position point should not be less than the end time of the lot production task on the front position point adjacent to the previous machine.

If there is no lot production task at the position point of the previous machine, then the left side of the inequality is a minimal negative value, and the right side is a positive value, and the inequality is constant. If there is no lot production task at the position point of the rear machine, then the right side of the inequality is a maximum positive value, and the left side is a normal positive value, and the inequality is constant; if there are lot production tasks at nonadjacent positions of the two machines, then *Z*(1 − *x*_*ijkt*_)=0. If the intermediate inventory from *k* to *k*′ is non-negative and the intermediate inventory from *k* − 1 to *k*′ is non-negative, then *Ze*_*ij*,*k*−1,*k*′*t*_+Z(1 − *e*_*ijkk*′*t*_)⟶*∞*, and the inequality is constant; if the intermediate inventory from *k* to *k*′ is less than 0 and the intermediate inventory from *k* − 1 to *k*′ is less than 0, or if the intermediate inventory from *k* to *k*′ is less than 0 and the intermediate inventory from *k* − 1 to *k*′ is non-negative, or if the intermediate inventory from *k* to *k*′ is non-negative and the intermediate inventory from *k* − 1 to *k*′ is less than 0, then the above three situations do not satisfy the assumption that there is a lot production task at the nonadjacent position points of the two machines, so the above three situations do not hold. The above analysis can be expressed by the following equation:

(9) $$\begin{array}{c} tstart_{ijkt}+q_{ij}x_{ijkt}+\lambda_{ij}u_{ijkt}\theta_{ij}x_{ijkt}-Z\left(1-x_{ijkt}\right)\leq tstart_{i,j+1,k't}+Z\left(1-x_{i,j+1,k't}\right)+Ze_{ij,k-1,k't}+Z\left(1-e_{ijkk't}\right),\forall i\in P,j\in S,j\neq jm,k\in N,k^{\prime }\in N,t\in T\text{.} \end{array}$$

If there is no lot production task at the position point of the previous machine, then the left side of the inequality is a minimal negative value, and the right side is a positive value, and the inequality is constant. If there is no lot production task at the position point of the rear machine, then the right side of the inequality is a maximum positive value, and the left side is a normal positive value, and the inequality is constant; if there are lot production tasks at nonadjacent positions of the two machines, then *Z*(1 − *x*_*ijkt*_)=0. If the intermediate inventory from *k* to *k*′ is less than the maximum ability limit and the intermediate inventory from *k* − 1 to *k*′is less than the maximum ability limit, then *Zv*_*ijk*,*k*′−1,*t*_+*Z*(1 − *v*_*ijkk*′*t*_)⟶*∞*, and the inequality is constant; if the incoming intermediate inventory from *k* to *k*′ exceeds the maximum ability limit and the incoming intermediate inventory from *k* − 1 to *k*′ exceeds the maximum ability limit, or if the incoming intermediate inventory from *k* to *k*′ exceeds the maximum ability limit and the incoming intermediate inventory from *k* − 1 to *k*′ is less than the maximum ability limit, or if the incoming intermediate inventory from *k* to *k*′ is less than the maximum ability limit and the incoming intermediate inventory from *k* − 1 to *k*′ exceeds the maximum ability limit, then the above three situations do not satisfy the assumption that there are lot production tasks at the nonadjacent positions of the two machines, so the above three situations do not hold. The abovementioned analysis can be expressed by the following equation:

(10) $$\begin{array}{c} tstart_{i,j+1,k^{\prime }t}-Z\left(1-x_{i,j+1,k^{\prime }t}\right)\leq tstart_{ijkt}+q_{ij}\alpha_{ijkt}+\lambda_{ij}u_{ijkt}\theta_{ij}x_{ijkt}+Z\left(1-x_{ijkt}\right)+Zv_{ijk,k^{\prime }-1,t}+Z\left(1-v_{ijkk^{\prime }t}\right),\forall i\in P,j\in S,j\neq jm,k\in N,k^{\prime }\in N,t\in T \end{array}$$

#### 3.2.5. System Disturbance

To provide optimal decisions for the process industry lot sizing and scheduling integrated optimization problem defined here, we can do this by optimizing equations (1)-(11) within a given time horizon. However, the solution of this approach works only if the perturbation of the system remains constant, which is not the case in real life. Therefore, this paper needs to develop flexible decision support models to find optimal operational decisions in dynamic environments.

In this paper, the system's disturbance is mainly divided into two parts: the uncertain market demand and the uncertain equipment production ability. Among them, the dynamic nature of customer demand leads to the uncertainty of future market demand, and the uncertainty of future market demand will directly lead to changes in the production plan of the process industry, which in turn affects the arrangement of lot sizing and scheduling in the next step. Therefore, how to convert the uncertain market demand in the future into a specific value has become the key for the process industry to formulate production plans. Driven by the industrial Internet, managers organize the historical order data processed by the workshop and upload it to the cloud platform for storage, which enables the system to analyze the demand trend of each product through chronological orders and then infer the market demand determined in the future.

Because this article aims to produce equal supply and demand, the sum of the lot production tasks of each product on the production line in the order in the last equipment should be consistent with the market demand for the product, which can be expressed by the following equation:

(11) $$\begin{array}{c} \underset{k\in P}{\sum }y_{ijkt}=d_{it},\forall i\in P,j=jm,t\in T. \end{array}$$

The uncertainty of demand mainly comes from the external market, and there are many influencing factors, such as the product's essential attributes, the market's overall economic development, customers' preferences, and a series of factors. However, the uncertain market demand dominated by the abovementioned factors cannot be controlled by manufacturing enterprises as the main production body. However, for the workshops within the enterprise, the uncertainty generated in the production process can be predicted and controlled. Among them, the production ability is a more typical one.

In this paper, production ability mainly refers to the maximum production ability of equipment in the process industry. In the actual processing process, the equipment will experience aging, wear, and even sudden failure with the increase of the production cycle. When the above conditions occur, the production ability of the equipment will change accordingly. Therefore, the production ability of the equipment is closely related to the failure rate. In order to better describe the relationship between the production ability of the equipment and the failure rate, this paper first introduces the failure rate distribution function, which can be described by the Weibull function, where *η*_*j*_ is the scale parameter and *m* is the shape parameter.

(12) $$\begin{array}{c} \varphi_{j}^{0}\left(t\right)=1-\exp\;\left(-{\left(\frac{t}{\eta_{j}}\right)}^{m}\right),t\in T. \end{array}$$

However, in the actual situation, according to the actual processing conditions, when the processing time of the equipment increases, the probability of its failure will significantly increase, which is expressed by the following equation:

(13) $$\begin{array}{c} \varphi_{j}\left(t\right)=\mu_{j}\cdot \varphi_{j}^{0}\left(t\right),t\in T. \end{array}$$

Among them, *φ*_*j*_(*t*) represents the failure rate distribution function of the equipment *j* processing the order *t* and *μ*_*j*_ represents the failure rate increment factor of the equipment *j*. In the early processing stage of the workshop, the original maximum production ability of the equipment is known, so when the equipment *j* fails in the processing order *t*, its production ability can be expressed by the following equation:

(14) $$\begin{array}{c} bb_{ijt}^{\max^{\prime }}=\left(1-\varphi_{j}\left(t\right)\right)bb_{ijt}^{\max},t\in T. \end{array}$$

where *bb*_*ijt*_^max^ represents the original maximum production ability of equipment *j* in order *t* and *bb*_*ijt*_^max′^ represents the existing maximum production ability of equipment *j* in order *t*. Here, we integrate equations (12)-(14) to obtain the following equation:

(15) $$\begin{array}{c} bb_{ijt}^{\max^{\prime }}=\left(1-\mu_{j}\cdot \left(1-\exp\;\left(-{\left(\frac{t}{\eta_{j}}\right)}^{m}\right)\right)\right)bb_{ijt}^{\max}\text{.} \end{array}$$

It can be seen from equation (15) that the calculation formula of the equipment failure rate changes with the shape parameters, so the calculation of the equipment production ability is an apparent nonlinear problem.

### 3.3. Total Completion Time Function and Total Production Cost Function

In the production scheduling problem of the process industry, if only the material network is considered, then the amount of intermediate inventory needs to be as small as possible, that is, within a reasonable range; and if it involves the production and manufacture of multiproduct processing, it is necessary to make the most of the design plan and reduce the number of handovers possibly. The problem that needs to be optimized in this paper is how to reduce the intermediate inventory generated in the material network and how to reduce the number of production switching times in production manufacturing when the total processing time is as small as possible in the division and scheduling of design lots.

For the enterprise, because each piece of equipment can only process one product at a time, workers also need to clean and adjust the equipment when production switching occurs on the equipment. Because of the relationship between the product purity label and chemical properties such as composition, the switching requirements between different labels are different. If it is switched from low-grade to high-grade, then the cleaning and adjustment requirements of the equipment will be correspondingly reduced, and the time and cost consumption will be relatively less. However, if it is switched from high-grade to low-grade, then the requirements for cleaning and adjustment of equipment will be significantly increased compared with the previous case. Switching from high to low requires more procedures and even needs to add cleaning materials. Therefore, when switching from high to low, time and cost consumption will increase significantly. To sum up, the optimization goal of the mathematical model of lot and scheduling integrated optimization in the process industry should be based on the time and cost of production switching and material transfer.

#### 3.3.1. Total Completion Time Function

From the perspective of time, the lot completion time of each product is composed of three parts, namely, the start time *tstart*_*ijkt*_ of the lot, the processing time of the material *λ*_*ij*_*θ*_*ij*_*u*_*ijkt*_*x*_*ijkt*_ in the lot, and the adjustment and cleaning time *q*_*ij*_*x*_*ijkt*_ brought by the product switching. Among them, the other two periods are determined by the lot, and the product switching time *q*_*ij*_*x*_*ijkt*_ can be regulated by the scheduling scheme to minimize the impact of this switching time on the total completion time. Therefore, the lot completion time of each product in each order can be expressed by the following equation:

(16) $$\begin{array}{c} tend_{ijkt}=tstart_{ijkt}+\lambda_{ij}\theta_{ij}u_{ijkt}x_{ijkt}+q_{ij}x_{ijkt.} \end{array}$$

In order to compare the optimal scheme of the system in each order, the first objective function can be obtained by selecting the shortest total processing time as the criterion, which is expressed by the following equation:

(17) $$\begin{array}{c} F_{1}=\min\;\left\{\max\;\left\{tstart_{ijkt}+\lambda_{ij}\theta_{ij}u_{ijkt}x_{ijkt}+q_{ij}x_{ijkt}\left|i\in P\right.\right\}\right\}\text{.} \end{array}$$

Among them, *tstart*_*ijkt*_ represents the lot start processing time of the product *i* at the position *k* of stage *j* in order *t*; *λ*_*ij*_*θ*_*ij*_*u*_*ijkt*_*x*_*ijkt*_ represents the lot processing time of the product *i* at the position *k* of stage *j* in order *t*; and *q*_*ij*_*x*_*ijkt*_ represents the time when the product *i* adjusts and cleans the equipment during the variety switching at the location of phase *j* in order *t*.

#### 3.3.2. Total Production Cost Function

From a cost perspective, different products in the same equipment will lead to different switching costs due to different purity labels, which can be expressed as *q*_*ij*_*prz*_*ij*_. However, the equipment does not switch varieties every time, and it needs to be defined by *x*_*ijkt*_. Therefore, the switching cost of each equipment in each order can be expressed by the following equation:

(18) $$\begin{array}{c} C_{zh}=\underset{i\in P}{\sum }\underset{k\in N}{\sum }\left(q_{ij}x_{ijkt}\times p{rz}_{ij}\right). \end{array}$$

In addition, when a particular lot of products is produced on adjacent equipment, it may not be ready for production and use, so intermediate inventory will be generated, and then inventory costs will be incurred. Therefore, the inventory cost of processing a product on each equipment in each order can be expressed by the following equation:

(19) $$\begin{array}{c} C_{kc}=\underset{k\in N}{\sum }\underset{k^{\prime }\in N}{\sum }\left(\left(\underset{0\leq kk\leq k}{\sum }y_{ij,kk,t}-\underset{0\leq kk\leq k^{\prime }}{\sum }u_{i,j+1,kk,t}\right)\cdot \mathrm{pr}k_{ij}\right). \end{array}$$

Therefore, when studying the process production control system proposed in this paper, the total production cost composed of switching cost and inventory cost needs to be used as another essential objective function to calculate the lot sizing and scheduling of multiproducts in each order.

(20) $$\begin{array}{c} F_{2}=\min\underset{i\in P}{\sum }\underset{j\in S}{\sum }\underset{k\in N}{\sum }\left(q_{ij}x_{ijkt}\times \mathrm{pr}z_{ij}+\underset{k^{\prime }\in N}{\sum }\left(\left(\underset{0\leq kk\leq k}{\sum }y_{ij,kk,t}-\underset{0\leq kk\leq k^{\prime }}{\sum }u_{i,j+1,kk,t}\right)\cdot \mathrm{pr}k_{ij}\right)\right). \end{array}$$

Among them, *q*_*ij*_*x*_*ijkt*_ represents the switching time generated when a particular lot of products in the order is switched in a particular stage, and it can multiply with the unit switching cost of this stage to obtain the corresponding switching cost. $\underset{k\in N}{\sum }\underset{k^{\prime }\in N}{\sum }\left(\left(\underset{0\leq kk\leq k}{\sum }y_{ij,kk,t}-\underset{0\leq kk\leq k^{\prime }}{\sum }u_{i,j+1,kk,t}\right)\cdot \mathrm{pr}k_{ij}\right)$ represents the intermediate inventory produced by a particular lot of products in the order in a certain stage, which can multiply with the unit inventory cost of this stage to obtain the corresponding inventory cost.

### 3.4. Model Predictive Control

MPC is a control algorithm that introduces feedforward effects based on process models and disturbance predictions, enabling the system to react in advance and to make appropriate changes to counteract the foreseen disturbances [31]. The implementation of the MPC algorithm mainly includes three processes: prediction model, rolling optimization, and feedback control. Among them, the system can predict the uncertain variables in the future through the prediction model to find the foreseeable disturbance, and after obtaining the predicted data, the system can respond to the disturbance through the optimization model to make the system update the decision to complete the optimization transmission into the next cycle. Because MPC uses optimization tools, it can naturally consider state and control constraints while finding the best controller. Furthermore, the rolling level approach and its predicted levels allow MPC to handle systems with complex dynamic behavior, making it more suitable for online implementation.

#### 3.4.1. Optimization Problem

In the process of rolling optimization of the model predictive control, the system requires that a nonlinear optimization problem be solved online at each sampling time to obtain a control effect. Therefore, the setting of the optimization problem is significant. It can be known from the previous literature [32, 33] that the optimization problem of MPC mainly revolves around two aspects, that is, reducing the cumulative prediction error as much as possible in the process of predictive control. It is the same as the cumulative control increment, and their proportion is equally significant; thus, we can calculate the optimized performance index of MPC as shown in the following equation:

(21) $$\begin{array}{c} minJ\left(k\right)=\frac{1}{2}\overset{N_{p}}{\underset{j=1}{\sum }}{\left(y_{k+j}-y_{k+j}^{sj}\right)}^{T}\left(y_{k+j}-y_{k+j}^{sj}\right)+\frac{1}{2}\overset{N_{u}}{\underset{j=1}{\sum }}\Delta u_{k+j}^{T}\Delta u_{k+j.} \end{array}$$

Among them, *N*_*p*_ and *N*_*u*_ are the prediction time domain and the control time domain, respectively, (*N*_*p*_ ≥ *N*_*u*_ > 0). *y*_*k*+*j*_ represents the predicted output value; *y*_*k*+*j*_^*sj*^ represents the actual output value; and Δ*u*_*k*+*j*_ represents the control increment and Δ*u*_*k*+*j*_=*u*_*k*+*j*_ − *u*_*k*+*j*−1_. In addition, the control variables must also meet certain constraints, namely, *u*_min_ ≤ *u*_*k*+*j*_ ≤ *u*_max_ and Δ*u*_min_ ≤ Δ*u*_*k*+*j*_ ≤ Δ*u*_max_.

In this paper, the prediction error is mainly proposed for the Elman neural network to predict the parameters related to market demand and equipment production ability; and the control increment is mainly proposed for screening out the optimal lot scheduling scheme in the dynamic optimization model. The system first trains the most suitable Elman neural network based on the minimum cumulative prediction error and then substitutes the parameters predicted by the Elman neural network into the optimization model so that the minimum cumulative control increment can be used as the rule to determine the noninferior solution set. The optimal scheduling scheme is sent back to the system to complete the model predictive control process.

#### 3.4.2. Status Feedback

After completing the entire model predictive control process with the MPC optimization problem as the goal, a new set of optimal scheduling schemes can be obtained, and the system needs to send back feedback on the status in time. In this article, the status feedback refers to the update of the scheduling scheme, and the update method is as follows:

(22) $$\begin{array}{c} u_{ijkt}=u_{ijk,t-1}+\Delta u_{ijk,t-1,t.} \end{array}$$

Among them, *u*_*ijkt*_ represents the new input lot that product *i* is allocated to the *k*th position onstage *j* in the predicted order *t*; *u*_*ijk*,*t*−1_ represents that product *i* is allocated in the known latest order *t* − 1 to the known input lot at the *k*th position onstage *j*; and ΔΔ*u*_*ijk*,*t*−1,*t*_ represents the change of the same lot between the predicted order *t* and the known latest order *t* − 1.

#### 3.4.3. MPC Implementation

**Figure 2:**
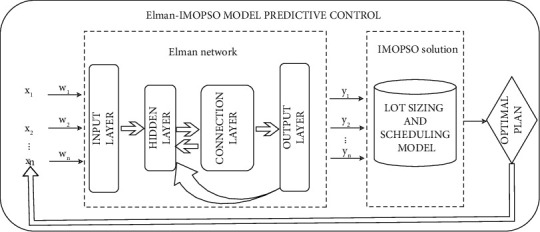
Model predictive control process implementation diagram.

It can be seen from the abovementioned analysis that the prediction link of the model predictive control is realized by the Elman neural network. After the Elman neural network learns from historical orders, it can continuously train the network based on the minimum cumulative prediction error and effectively predict the bidirectional uncertain market demand and the production ability to output the product demand variables and the parameter variables related to the failure rate calculation. The optimization model is input, and the IMOPSO algorithm is used to solve the model, and the optimal decision-making scheme considering the foreseeable disturbance is obtained based on the minimum change and is sent back to the system. So far, the model predictive control for integrated optimization of lot and scheduling in the process industry with bidirectional uncertainties has been formed, and its specific implementation process is shown in Figure 2:

In the implementation process of the entire model predictive control, the decision optimization model can find the control strategy that minimizes the objective function while responding to the predicted data. Then, the system sends back the updated strategy in time for feedback correction. Therefore, the decision optimization model is the core link of MPC implementation. Here, we refer to reference [34] for a decision-making optimization model for the integration of lot sizing and scheduling in the process industry, that is, the objective function is to minimize the total completion time given by equation (23) and minimize the production cost given by equation (24); equations (25)-(33), respectively, form the constraints of material network and manufacturing; and equations (34) and (35), respectively, calculate the uncertain market demand and production ability, which are represented by this form the constraints of the decision optimization model, which are expressed explicitly as follows:

(23) $$\begin{array}{c} F_{1}=\min\;\left\{\max\;\left\{tstart_{ijkt}+\lambda_{ij}\theta_{ij}u_{ijkt}x_{ijkt}+q_{ij}x_{ijkt}\left|i\in P\right.\right\}\right\}\text{.} \end{array}$$

(24) $$\begin{array}{c} F_{2}=\min\underset{i\in P}{\sum }\underset{j\in S}{\sum }\underset{k\in N}{\sum }\left(q_{ij}x_{ijkt}\times \mathrm{pr}z_{ij}+\underset{k^{\prime }\in N}{\sum }\left(\left(\underset{0\leq kk\leq k}{\sum }y_{ij,kk,t}-\underset{0\leq kk\leq k^{\prime }}{\sum }u_{i,j+1,kk,t}\right)\cdot \mathrm{pr}k_{ij}\right)\right). \end{array}$$

(25) $$\begin{array}{c} \underset{k\in N}{\sum }y_{ijkt}=\underset{k\in N}{\sum }u_{ijkt}\theta_{ij},\forall i\in P,j\in S,t\in T \end{array}$$

(26) $$\begin{array}{c} x_{ijkt}Bb_{ijt}^{\min}\leq u_{ijkt}\leq x_{ijkt}Bb_{ijt}^{\max^{\prime }}\forall i\in P,j\in S,k\in N,t\in TI_{ijkk^{\prime }t}=I_{i,j-1,k^{\prime \prime }kt}+\underset{0\leq kk\leq k}{\sum }y_{ij,kk,t}-\underset{0\leq kk\leq k^{\prime }}{\sum }u_{i,j+1,kk,t},\forall i\in P,j\in S,j\neq jm,k\in N,k^{\prime }\in N,k^{\prime \prime }\in N,t\in T \end{array}$$

(27) $$\begin{array}{c} 0\leq I_{ijkk^{\prime }t}\leq I_{ij}^{\max},\forall i\in P,j\in S,k\in N,k^{\prime }\in N,t\in T\text{.} \end{array}$$

(28) $$\begin{array}{c} \underset{i\in P}{\sum }x_{ijkt}\leq 1,\forall j\in S,k\in N,t\in T \end{array}$$

(29) $$\begin{array}{c} \underset{i\in P}{\sum }x_{ijkt}\geq \underset{i\in P}{\sum }x_{ij,k+1,t},\forall j\in S,k\in N,k\neq km,,t\in T \end{array}$$

(30) $$\begin{array}{c} {\text{tstart}}_{ij,k+1,t}\geq {\text{tstart}}_{ijkt}+q_{ij}x_{ijkt}+\lambda_{ij}u_{ijkt}\theta_{ij}\;x_{ijkt},\forall i\in P,j\in S,k\in N,k\neq km,t\in T \end{array}$$

(31) $$\begin{array}{c} {tstart}_{i'jkt}+q_{i'j}x_{i'jkt}+\lambda_{i'j}u_{i'jkt}\theta_{i'j}x_{ijkt}-Z\left(1-x_{i'jkt}\right)+T_{i'ijt}x_{ij,k+1,t}\leq tstart_{ij,k+1,t}\;,\forall i\in P,i^{\prime }\in P,j\in S,k\in N,t\in T\text{.} \end{array}$$

(32) $$\begin{array}{c} {\text{tstart}}_{ijkt}+q_{ij}x_{ijkt}+\lambda_{ij}u_{ijkt}\theta_{ij}x_{ijkt}-Z\left(1-x_{ijkt}\right)\leq tstart_{i,j+1,k't}+Z\left(1-x_{i,j+1,k't}\right)+Ze_{ij,k-1,k't}+Z\left(1-e_{ijkk't}\right),\forall i\in P,j\in S,j\neq jm,k\in N,k^{\prime }\in N,t\in T \end{array}$$

(33) $$\begin{array}{c} tstart_{i,j+1,k^{\prime }t}-Z\left(1-x_{i,j+1,k^{\prime }t}\right)\leq tstart_{ijkt}+q_{ij}\alpha_{ijkt}+\lambda_{ij}u_{ijkt}\theta_{ij}x_{ijkt}+Z\left(1-x_{ijkt}\right)+Zv_{ijk,k^{\prime }-1,t}+Z\left(1-v_{ijkk^{\prime }t}\right),\forall i\in P,j\in S,j\neq jm,k\in N,k^{\prime }\in N,t\in T \end{array}$$

(34) $$\begin{array}{c} \underset{k\in P}{\sum }y_{ijkt}=d_{it},\forall i\in P,j=jm,t\in T \end{array}$$

(35) $$\begin{array}{c} bb_{ijt}^{\max^{\prime }}=\left(1-\mu_{j}\left(1-\exp\;\left(-{\left(\frac{t}{\eta_{j}}\right)}^{m}\right)\right)\right)bb_{ijt}^{\max}\text{.} \end{array}$$

#### 3.4.4. Algorithm Design

The artificial neural network can simulate the way of human brain neural network processing and memory information so that it has the ability of large-scale parallel processing and highly nonlinear problem processing—a series of dynamic complex data. In the existing research, scholars often use the BP neural network to solve it. However, BP neural network is a feedforward network that takes a long time to train and quickly falls to a local minimum. The Elman neural network is a typical dynamic feedback neural network. Based on the basic structure of the BP network, a connection layer is added to achieve the purpose of memory so that it can internally feedback, store, and use the output information of the past time. It can realize the model of the static system and the mapping of the dynamic system and directly reflect the system's dynamic characteristics. It is better than the BP neural network in terms of computing power and network stability. Since the production system studied in this paper learns from the existing external market demand and internal equipment failure data, it predicts the future order demand and production ability. The production system is rescheduled according to the predicted data, which has specific adaptive time-varying characteristics. Therefore, we use the Elman neural network for prediction. The specific structural diagram is as follows.

**Figure 3:**
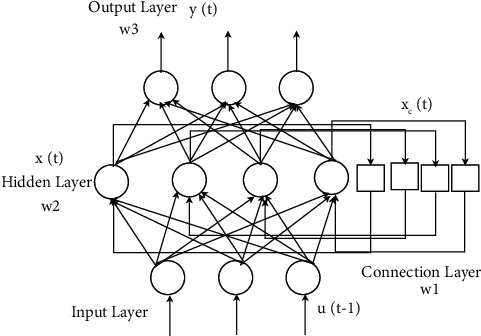
Schematic diagram of the Elman neural network structure.

As shown in Figure 3, the structure of the Elman neural network is divided into four layers: the input layer, the hidden layer, the connection layer, and the output layer. Among them, the input layer mainly plays the role of incoming data. The hidden layer is mainly used to connect the feedback of the output layer and the receiving layer and affects the input data through the adjustment of the weights; the receiving layer realizes the delayed input function of the data, the output layer. Then, the hidden layer's data output is linearly weighted. In the Elman neural network, the calculation formula of each layer is shown in the following equations:

(36) $$\begin{array}{c} x_{c}\left(t\right)=x\left(t-1\right) \end{array}$$

(37) $$\begin{array}{c} x\left(t\right)=f\left(w_{1}x_{c}\left(t\right)+w_{2}\left(u\left(t-1\right)\right)\right)\text{.} \end{array}$$

(38) $$\begin{array}{c} y\left(t\right)=g\left(w_{3}x\left(t\right)\right)\text{.} \end{array}$$

Among them, *u* is the output vector of the hidden layer, *y* is the input vector of the input layer, *x* is the output vector of the hidden layer, and *x*_*c*_ is the output vector of the connection layer. *w*_1_ is the weight vector from the connection layer to the hidden layer, *w*_2_ is the weight vector from the input layer to the hidden layer, and *w*_3_ is the weight vector from the hidden layer to the output layer. The activation functions of the hidden layer and output layer are f(t) and g(t), respectively. Generally speaking, *f*(*t*) and *g*(*t*) are the “tansig” function and the “purelin” function, respectively, and the training algorithm adopts the “traingdx” algorithm. Among them, the expression formulas of “tansig” function and the “purelin” function are as follows:

(39) $$\begin{array}{c} f\left(t\right)=\frac{2}{1+e^{-2t}}-1\text{.} \end{array}$$

(40) $$\begin{array}{c} g\left(t\right)=t\text{.} \end{array}$$

It can be seen from the recursion of equations (37)-(40) that the data of the output layer of the neural network is calculated by relying on the nonlinear function composed of *f*(*t*) and *g*(*t*), that is, the calculation of the hidden layer and the output layer. The formulas are shown in equations (41)-(42).

(41) $$\begin{array}{c} x\left(t\right)=\frac{2}{1+e^{-2\left(w_{1}x\left(t-1\right)+w_{2}\left(u\left(t-1\right)\right)\right)}}-1\text{.} \end{array}$$

(42) $$\begin{array}{c} y\left(t\right)=\frac{2w_{3}}{1+e^{-2\left(w_{1}x\left(t-1\right)+w_{2}\left(u\left(t-1\right)\right)\right)}}-1\text{.} \end{array}$$

After the complete neural network is obtained, the system will transfer the predicted data to the optimization model, obtain the updated plan by solving, and send it back to the system as the reference input value for the next order cycle of the neural network. When solving the past mathematical model of the scheduling problem, particle swarm optimization is often widely used due to its advantages, such as easy implementation, high precision, and fast convergence. However, there is more than one objective function in the lot sizing and scheduling integrated model established in this paper, so here we use the MOPSO algorithm to solve it. In order to further improve the effectiveness of the algorithm and reduce the computational complexity, this paper integrates the multiobjective evolution idea with problem decomposition and optimization as the core into the particle swarm iteration mechanism and names it IMOPSO for use. In summary, the model predictive control algorithm proposed in this paper is designed as follows:

*Step1*. Substitute the requirements of each product (or equipment failure parameters) in the historical orders as the existing data into the input layer of the Elman neural network. Because there is a specific time series relationship between the historical orders studied in this paper, the latter in the adjacent order is substituted into the network as the output layer corresponding to the former input layer.
*Step2*. Divide the dataset in the network into the training set, validation set, and test set and normalize the data; set a series of initial parameters for the algorithm, such as the maximum number of iterations required by IMOPSO and population size. Randomly assign each subproblem assignment weight vector.
*Step3*. Obtain the number of nodes in the input and output layers. The number of nodes in the hidden layer can be calculated as shown in equation (43). Among them, the number of nodes in the connection layer and the hidden layer is the same and generally *ζ* takes an integer between 1 and 10.
  (43) $$\begin{array}{c} hiddennum=\sqrt{\left(inputnum+outputnum\right)}+\zeta . \end{array}$$
*Step4*. Randomly generate the connection weights *w*_1_, *w*_2_, *an* *d* *w*_3_ from the connection layer to the hidden layer, the input layer to the hidden layer, and the hidden layer to the output layer, respectively, and calculate the output value of each layer according to the formulas given in equations (41) and (42).
*Step5*. Use the gradient descent method as the learning algorithm of the Elman neural network, and the error can be calculated by using equation (31). Among them, *y*(*t*) represents the predicted output value and *y*^*sj*^(*t*) represents the actual output value.
  (44) $$\begin{array}{c} E=\frac{{\left(y\left(t\right)-y^{sj}\left(t\right)\right)}^{T}\left(y\left(t\right)-y^{sj}\left(t\right)\right)}{2} \end{array}$$
*Step6*. If the error does not meet the accuracy, calculate the partial derivatives of the error function *E* to different weights, respectively, and then adjust the weights according to the gradient descent method in the direction of negative gradient, that is, Δ*w* = −*η∂E*/*∂w*, and then adjust the weights. Go to Step4 to continue the calculation, where *η* is the learning rate; and if the error meets the accuracy, go directly to Step7.
*Step7*. The Elman neural network predicts the historical order data (equipment failure data) to initialize the population and calculates the fitness value of the particle to find the individual optimal particle *Pbest*. The *g*^*te*^ value of each particle is obtained according to the Chebyshev method (as shown in equation (45)).
  (45) $$\begin{array}{c} g^{te}\left(x\left|\lambda^{j},z^{*}\right.\right)=\max_{1\leq i\leq m}\;\left\{\lambda_{i}^{j}\left|f_{i}\left(x\right)-z_{i}^{*}\right|\right\}\text{.} \end{array}$$
*Step8*. Initialize the Pareto optimal solution set and find nondominated solutions in the particle swarms that meet the requirements and then put them into the Pareto optimal solution set.
*Step9*. For each population, a roulette method randomly selects a particle as the global optimal particle *Gbest*.Use the velocity and position update equations (46) and (47) to update each particle in the population and continue to adjust the updated particle population by substituting the constraints. If a better particle is produced, the individual optimal particle *Pbest* is updated.
  (46) $$\begin{array}{c} v_{i\;d}=\omega \cdot v_{i\;d}+c_{1}\cdot r_{1}\left(p_{i\;d}-x_{i\;d}\right)+c_{2}\cdot r_{2}\left(p_{g\;d}-x_{i\;d}\right)\text{.} \end{array}$$
  (47) $$\begin{array}{c} x_{i\;d}=v_{i\;d}+x_{i\;d}. \end{array}$$
*Step10*. Randomly select two particles in the neighbors of each particle to cross to obtain a new particle *y* and substitute it into the constraints to repair. Compare the objective function value corresponding to the particle with the reference point *z* and select a smaller value to substitute in *z* to update.
*Step11*. Calculate the *g*^*te*^ values obtained when the particle *y* corresponds to different *λ* values in the neighbors of the original particle by using equation (45). If the *g*^*te*^ value of the particle *y*is higher than the *g*^*te*^ value of the current particle during the calculation process, the particle *y* is assigned to the current particle for updating, and *Pbest* is updated accordingly.
*Step12*. Recalculate the nondominated solutions in the updated population and put them into the Pareto optimal solution set. First, check whether there are still dominant solutions in the nondominated solution set and, if so delete the dominant solutions. Furthermore, it is necessary to check whether the number of nondominated solutions reaches the maximum number of nondominated solution populations, and if it exceeds the number, the redundant part should be deleted.
*Step13*. Repeat Step9 to Step12 when the number of iterations is less than the maximum. When the maximum number of iterations is reached, the iterative output results stop, the output results screen according to the principle of controlling the cumulative increment to be the smallest, and the best solution after screening is passed back to the neural network.

From the abovementioned steps, it is not difficult to find that the Elman neural network is a nonlinear model and whether the final output of each layer is obtained after calculation or the correction process of the weights in the network. It can be seen that the model predictive control problem proposed in this paper based on the Elman neural network should be an optimal control scheme for nonlinear systems.

## 4. Simulation Analysis

Based on the data of a customized chemical enterprise with two production lines of 10,000 tons and one 1,000-ton production line in Shenyang, this paper simulates and establishes a process enterprise's production control system that can quickly respond to dynamic market demands. Inside the system is an assembly line with an annual output of 10,000 tons, which includes three production stages: the first lipidation reaction, the second lipidation reaction, and the polycondensation reaction. There are different reactors on the assembly line, and each reactor also has an ability limit: the upper limit is the maximum ability of the reactor, which is related to the type and specification of the reactor, and the lower limit is determined by the reaction conditions and technical requirements. Due to the different abilities of different reactors in the assembly line, their single production lots are not equal. In addition, when the product variety switches, the reactors in the three different production stages need to be cleaned and other related work needs to be performed, which increases the production time of the entire variety process.

In order to realize the model predictive control process of integrated optimization of lot sizing and scheduling in the process industry, the basic data of the enterprise is listed in detail here. Among them, Table 2shows the basic parameter settings of the chemical company's process production, and Tables 3 and 4 show the product demand of the company's historical orders within half a year and the relevant parameter data for calculating equipment failure rates. In order to facilitate the calculation in the environment of maintaining the nonlinear system, in this paper, the shape parameters involved in the calculation formula of the equipment failure rate are all 2. In addition, Tables 5-7 also list the lot scheduling schedules for different products at various stages in each order cycle.

**Figure 4:**
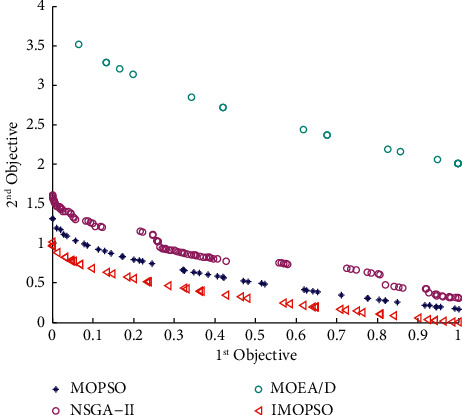
Frontier comparison diagram of the four algorithms under the ZDT1 function.

**Figure 5:**
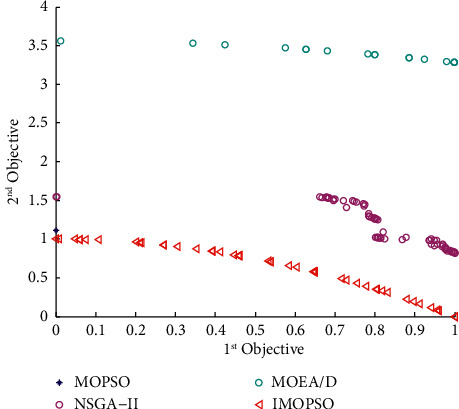
Frontier comparison diagram of the four algorithms under the ZDT2 function.

**Figure 6:**
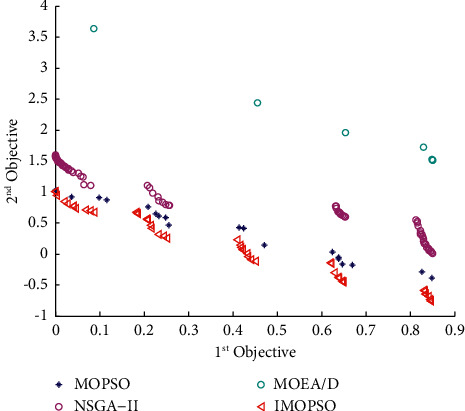
Frontier comparison diagram of the four algorithms under the ZDT3 function.

According to Section 3.4.3, the model predictive control algorithm proposed in this paper comprises the Elman neural network, the optimization model, and the IMOPSO algorithm. Among them, the selection of the Elman neural network has been explained in Section 3.4.4. However, the effectiveness of the IMOPSO algorithm and the optimization model built in this paper have not been proved. First, to verify the effectiveness of the IMOPSO algorithm, we introduce the ZDT series of test functions and set the population size and the number of iterations to 100 (for the MATLAB program, see https://github.com/cong0420/model-predictive-control/blob/main/ZDT%20TEST.zip), respectively. We compared IMOPSO with the most common MOPSO, NSGA-II, and MOEA/D algorithms for solving multiobjective scheduling models in the same initial population and iterative environment. The specific results are shown in Figures 4-6:

It can be seen from the abovementioned comparison that no matter under which standard test function is used, the Pareto frontier obtained by the IMOPSO algorithm is not only always at the forefront but also has the fastest convergence speed. In addition to the convergence, we can see from Figures 4–6 that the distribution of results obtained by the IMOSPO algorithm is also overall better. Therefore, the IMOPSO algorithm has particular effectiveness.

Second, to further prove the effectiveness of the optimization model established in this paper, considering material network and manufacturing decision-making, this paper compares three situations. One is a global method that comprehensively considers material network and manufacturing, one is a decentralized method that only considers manufacturing, and the other is a decentralized method that only considers material network, and they are named as case 1, case 2, and case 3, respectively. In case 1, the decision-making optimization model should consider the limited intermediate inventory in the material network and the minimum number of switches in manufacturing based on the minimum total completion time and total production cost, as shown in the mathematical model presented in Section 3.4.5. In case 2, considering only the manufacturing problem, it is necessary to reduce the number of switches in the production process as much as possible. It does not care about the inventory cost, so the upper limit of the maximum inventory should be calculated based on the original mathematical model. In case 3, considering only the material network problem, it is necessary to reduce the amount of intermediate inventory as much as possible. It does not care about the number of switches in the production process. Therefore, based on the original mathematical model, the link of calculating the switching cost should be deleted from the minimization of the second objective function.

After obtaining the division of different cases, we substitute the basic data in Table 2 and the data about order 24 in Tables 3 and 4 into the scheduling decision optimization model of the three cases and use the IMOPSO algorithm to carry out the three models. Solve to obtain the scheduling implementation plan after the decision optimization model responds to the forecast data. In order to objectively compare the calculation conditions of each target in different situations, this paper sets the population size and the number of iterations to 100, respectively, and uses the same initial population generated randomly to be substituted into different situations for iterative solutions. Here, we use the MATLAB software to randomly run the program ten times and find a set of results with more obvious comparisons from the ten sets of running results for observation (for the MATLAB program, see https://github.com/cong0420/model-predictive-control/blob/main/MODEL123.zip).

**Figure 7:**
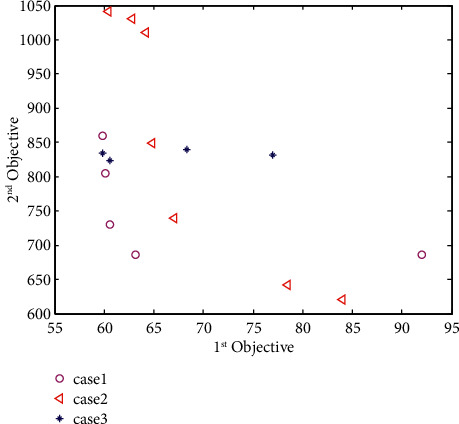
Comparison diagram of the Pareto frontiers in three cases.

As can be seen from Figure 7, the noninferior solution set obtained by case 1 is much better than the noninferior solution set obtained by case 2 and case 3. Among them, the total completion time of the three cases is not very different, and most of the schemes are between 60 and 85. From the perspective of total production cost, the total production cost of the solution obtained by case 1 is mainly concentrated between 600 and 850; the total production cost of the solution obtained by case 2 is mainly concentrated between 600 and 1050; and the total production cost of the solution obtained by case 3 is primarily concentrated in between 800 and 850, and the objective function values of each scheme under different circumstances are shown in Table 8.

In order to better observe the impact of different situations on the overall shop scheduling scheme, we analyze the switching cost and the inventory cost separately in the total production cost. As can be seen from Table 9, case 1, which must be considered at the same time, has the lowest switching cost; case 3, which only considers the intermediate inventory and does not consider the switching cost, has the most switching cost and only considers the switching cost. The switching cost obtained in case 2 without considering the intermediate inventory is between the two.

As can be seen from Table 10, the inventory cost obtained in case 3 is the least, which only considers the intermediate inventory quantity and does not consider the switching cost. Moreover, the inventory cost obtained in case 2 is the highest, which only considers the switching cost and does not consider the intermediate inventory quantity. The inventory cost obtained in case 1, which must be considered simultaneously, is located between the two.

It can be seen that whether from the perspective of the total objective function value or from the perspective of the total production cost divided into switching costs and inventory costs, the process industry lot process established in this paper takes into account the material network and production manufacturing. Overall, the scheduling model is better than the mathematical model that only considers the material network and production manufacturing. Therefore, the model established in this paper is an effective decision-making optimization model and an essential link in the implementation of model predictive control.

So far, the effectiveness of the IMOPSO algorithm and the dynamic optimization model established in this paper have been proved, and then the model predictive control will be implemented. First, from the perspective of learning control, when the prior knowledge cannot be fully obtained, it cannot be completed by classical dynamic programming alone. At this time, it is necessary to design a controller to estimate unknown information. The controller can recognize and process the state change of the controlled object and the change of the external environment based on its learning ability and can continuously learn and improve according to its characteristics to adapt to the changes of the controlled object. In this paper, the estimated unknown information link of the controller corresponds to the data prediction link in the model predictive control—the Elman neural network—so its construction is significant. First, we set the maximum number of iterations to 10000 and the learning rate and training target minimum error to be 0.1 and 0.00001, respectively.

When predicting the demand quantity of each product in the future order, according to the data in Table 3, there are four products in total. Because the neural network needs to learn the sequence relationship in the adjacent orders, the latter of the adjacent orders needs to be the output data of the former order, so the number of nodes in the input layer and the output layer should be four, respectively. In addition, we divide the first 70% of the order into the training set, 15% into the validation set, and the last 15% into the test set and then normalize the divided data to [ 0,1]. In order to determine the optimal number of nodes in the hidden layer (succession layer), this paper uses the following pseudocode for calculation:

  for a = 1:10
   hiddennum = fix(sqrt(inputnum + outputnum))+*a*; net = newelm(inputnum, outputnum,hiddennum,{“tansig”, “purelin”}, “traingdx”);
   net.trainParam.epochs = 10000; net.trainParam.lr = 0.01; net.trainParam.goal = 0.00001;
   net = train(net,inputn, outputn); an = sim(net,inputn); mse11 = mse(outputn,an);
   if mse11<1*e*+05
    hiddennum_best = hiddennum;
    break;
   end
  end

By substituting the data in Table 3 into the calculation, it can be obtained that the optimal number of nodes in the hidden layer (succession layer) is 3. Therefore, when predicting the demand of products, the input layer, hidden layer, successor layer, and output layer of the Elman neural network are, respectively, [4, 3, 3, 4]. Similarly, when predicting the relevant parameter variables of the failure rate of the equipment, we substitute the data in Table 4 into the calculation according to the same process, and we can obtain the input layer, hidden layer, succession layer, and output of the Elman neural network under different equipment failure rate parameters. The layers are [3, 3, 3, 3].

After the Elman neural network constructs, the computer can learn the equipment according to the chronological order. This paper uses MATLAB for programming. Taking the market demand as an example, we substitute the data in Table 3 into the constructed Elman neural network whose input layer, hidden layer, successor layer, and output layer are [4, 3, 3, 4], respectively, and the pass “trainingdx” function of the adaptive learning algorithm with momentum term starts training. In the training process, the number of iterations is set to 200, and the network is continuously trained based on the principle of the minimum accumulated error until the most suitable network is found within the iteration range for the next prediction. Here, we randomly run the program ten times (for the MATLAB program, see https://github.com/cong0420/model-predictive-control/blob/main/Elman%20neutral%20network.zip). Although the algebra of iterative termination in each running process may not be consistent, the prediction results are consistent and stable. We randomly select a group of Elman neural network models that have been successfully learned after 118 iterations, and the cumulative minimum error is 2.7782.

**Figure 8:**
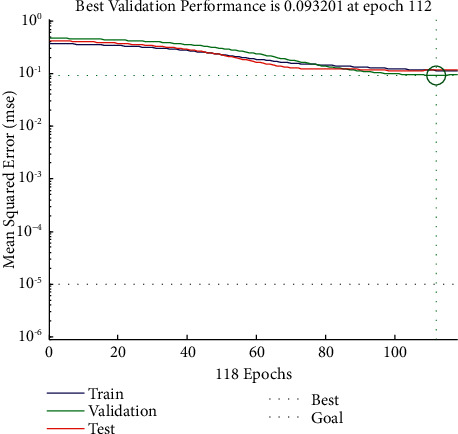
Change trend diagram of the Elman neural network mean square error on market product demand.

Although the mean square errors of the training set and the test set at the start and stable times are almost the same, the convergence speed of the test set is significantly faster than that of the training set. Compared with the training and test sets, the validation set has the largest mean square error at the beginning and the smallest mean square error at the stable time. Among them, at the 112th generation, it reaches a steady state, and its optimal mean square error value is 0.0932. As can be seen from Figure 8, although the numerical variation interval of the validation set is the largest, its convergence speed is relatively slow compared to the other two data sets. Therefore, we can conclude that in the Elman neural network constructed this time about market product demand, when iterating to the 112th generation, the change curves of each dataset can obtain their stable states, and their convergence speed is changed from fast to low: test set > training set > validation set.

**Figure 9:**
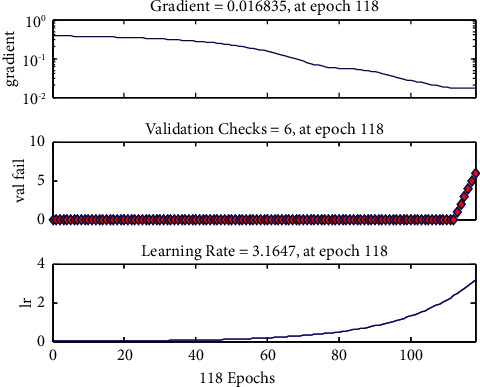
Gradient, validity test, and learning rate change trend diagram of the Elman neural network on market product demand.

As can be seen from Figure 9 that during the training process, the gradient decreases slowly, the learning rate increases exponentially, and the system judges that the output error of the validation set increases for six consecutive tests when it iterates to 118 generations. It shows that the error of the training set is no longer reduced so that the training stops. When the training is stopped, the gradient is 0.0168 and the learning rate is 3.1647.

**Figure 10:**
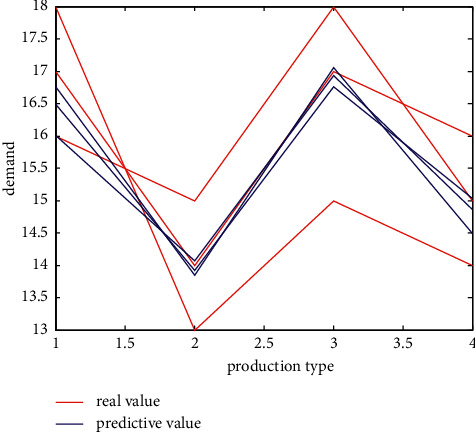
Comparison diagram of the predicted value of the test set and the actual output value of the Elman neural network on the market product demand.

Besides, this study also plots the predicted value in the test set against the actual output value. From Figure 10, we can see that the trend of the predicted values of each data group in the test set is similar. The error of the trend of the actual output is almost the same, which shows that the neural network has effectively learned the laws in the historical data, that is, the neural network. No matter what kind of input data is received, the predicted value can be predicted according to this rule, and the numerical result will not have a big difference from the actual data, and it has certain reliability.

Therefore, we substitute the product demand in order 24 into the professional Elman neural network of market demand. It can predict the demand for products in the following order. Similarly, the failure rate growth factor and the scale of the three pieces of equipment can be obtained. Parameters and the specific results are shown in Tables 11 and 12.

According to the description in Section 3.2.5, product requirements can be directly brought into equation (11) when calculating the production ability of each piece of equipment. It is necessary to bring the relevant parameters of the failure rate of each corresponding equipment production ability into Equation (15) calculation. As a result, the two-way uncertain product demand and production ability learned by the neural network can be transformed into definite values and integrated into the decision-making optimization model with subconstraints. So far, the forecast data obtained by Elman neural network and the decision calculated by the optimization model have been obtained.

However, it can be seen from Figure 7 that the optimization model obtains a noninferior solution set, so as to find the most suitable scheduling scheme among many nonsplitting solutions; thus, we must adopt the second criterion in the MPC optimization problem. Even if the cumulative control increment is as tiny as possible, in this paper, the cumulative lot change in the new scheme is also as tiny as possible. Therefore, we input the essential data in Tables 2-4 and the predicted data obtained in Tables 11 and 12 into the optimization model, set the population size and the number of iterations to 400, and use the IMOPSO algorithm to solve them. Based on the minimum cumulative control increment principle, the system selects the solutions in the noninferior solution set to obtain the optimal solution and sends it back to the system as the input reference value of the following order cycle of the neural network. We randomly ran the program ten times (for the MATLAB program, see https://github.com/cong0420/model-predictive-control/blob/main/simulation.zip), selected a group with noticeable results, and plotted the scheduling schemes before and after the implementation of MPC into Gantt charts and displayed them as follows.

**Figure 11:**
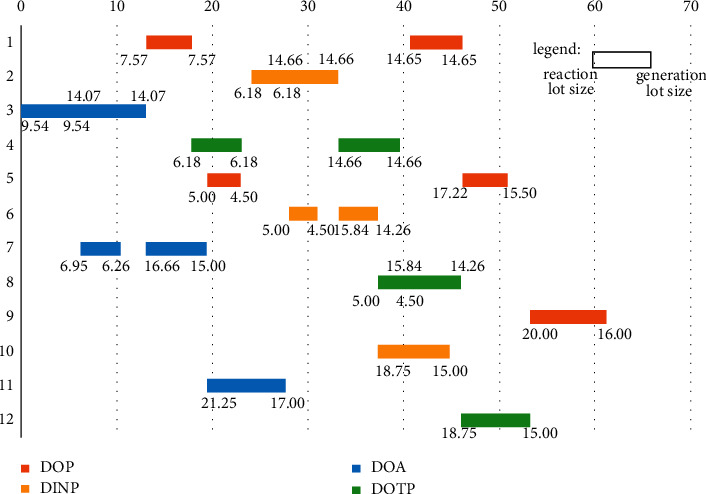
Gantt chart of scheduling diagram before model predictive control implementation.

**Figure 12:**
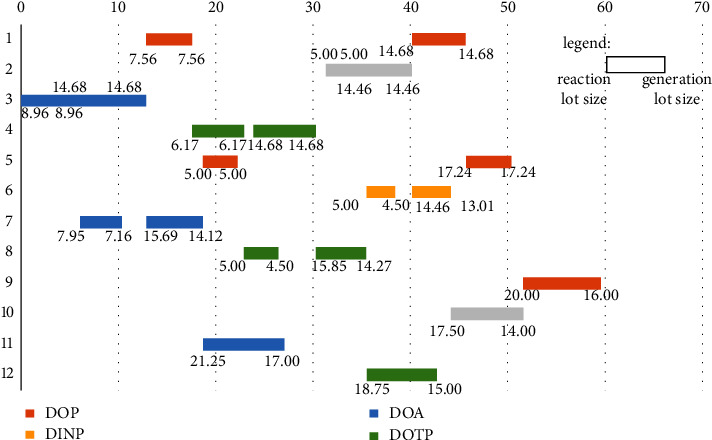
Gantt chart of scheduling diagram after model predictive control implementation.

It can be seen from Figures 11 and 12 that the values in the lower left and right corners of each lot correspond to the reaction volume and the generation volume of the lot, respectively. Among them, the material conversion rate on each piece of equipment is entirely consistent with the data in Table 2, and the intermediate inventory on two adjacent equipment does not exceed the maximum limit, and there is no time conflict between lots in the entire scheduling scheme. Through the comparison, it can be seen that through the model predictive control, the system can respond to the market demand and product production ability promptly and adjust the scheduling plan reasonably.

In order to better show the stability of the model predictive control algorithm established in this paper, here we introduce the idea of steady-state error and use it as a criterion to measure the controller's stability. The so-called steady-state error refers to the deviation that occurs when the system is disturbed and rebalanced. In this paper, it refers to the lot error of each lot before and after the implementation of model predictive control and it can be calculated by equation (48):

(48) $$\begin{array}{c} steady_{state_{error}}=\frac{\left(value_{new}-value\right)}{\left(value/100\right)}\text{.} \end{array}$$

Among them, *value*_*new*_ represents the lot after the implementation of the model predictive control, and the *value* represents the lot before the implementation of the model predictive control. The specific results of the steady-state errors of each lot before and after the implementation of model predictive control are shown in Table 13:

From Table 13, it can be seen that the steady-state error of 91.7% of the data before and after the model predictive control is controlled by ±10%. Furthermore, 66.7% of the steady-state error of the data is controlled by ±1%. Although a few data with relatively large steady-state errors due to the limitation of the equipment's capabilities occasionally appear in the table, they will not exceed ±20% of the original known data, which indicates that the controller can operate within a reasonable range. It is thus better to reduce system fluctuations. Besides that, through the comprehensive analysis of the data in Table 13 and the drawn Gantt chart, it can be found that the lot sizing before and after the implementation of model predictive control does not fluctuate wildly, and the overall scheduling scheme is orderly and reasonable. Therefore, in summary, the steady-state errors obtained in each lot before and after the implementation of the model predictive control are generally good, which shows that the model predictive control algorithm has good stability.

## 5. Conclusions

In the actual situation of uncertain market demand and production ability, this paper proposes a dynamic model for integrated optimization of lot sizing and scheduling in the process industry and implements it with MPC. The key to MPC implementation lies in predicting disturbances and optimizing the decision-making optimization model. First of all, regarding the prediction of disturbances, this paper uses the Elman neural network to learn the relevant variables of the demand quantity of each product in chronological order and the failure rate of the computing equipment to realize the scientific prediction of the target variable in the next cycle of orders in the future. Second, regarding the establishment of the decision-making optimization model, this paper not only considers the bidirectional uncertainty of market demand and production ability but also considers the process industry's material network and production manufacturing process and uses the IMOPSO algorithm to solve the problem. It can reduce costs and shorten the processing time by meeting the diverse needs of customers. So far, a complete model predictive control consisting of the Elman neural network, the optimization model, and the IMOPSO algorithm is formed. Finally, the model predictive control process is realized by example simulation. The effectiveness of the IMOPSO algorithm and the optimization model involved in the process are proved. In addition, this paper also analyzes the operation results combined with the scheduling Gantt chart from the perspective of scheduling arrangement and finds that the whole scheme not only satisfies the constraints but also has good production scheduling and specific stability.

Through model predictive control, the production system can react in advance and make appropriate changes to offset foreseeable disturbances, resulting in lot sizing and scheduling schemes that consider bidirectional uncertainty, improving the overall robustness of the system. For the process industry, the realization of the model predictive control is beneficial for enterprises to deal with various environments of uncertainty and is conducive to the process industry for further optimization of the lot sizing and scheduling of multiproduct production. In future work, we also need to introduce a more effective heuristic algorithm and integrate it into the Elman neural network to obtain a more efficient neural network learning mechanism.

## Figures and Tables

**Table 1:** Nomenclature

Index/set	Explanation
*i* ∈ *P*	Product
*j* ∈ *S*	Stage
*k* ∈ *N*	Position
*t* ∈ *T*	Order
Symbol	Explanation
x_ijkt_	It represents whether the product i is processed at the kth position onstage j in order t, if it is 1, otherwise it is 0
*u*_*ijkt*_	It represents the input lot of production that product *i* is assigned to the *kth* position onstage *j* in order *t*
*y*_*ijkt*_	It represents the output lot of production that product *i* is assigned to the *kth* position onstage *j* in order *t*
*Bb*_*ijt*_^max^	It represents the maximum limit for processing lots of product *i* onstage *j* in order *t*
*Bb*_*ijt*_^min^	It represents the minimum limit for processing lots of product *i* onstage *j* in order *t*
*θ*_*ij*_	It represents the conversion rate of product *i* processing lot onstage *j*
*tstart*_*ijkt*_	It represents the lot start processing time for the product *i* to be assigned to the *kth* position onstage *j* in order *t*
*tend*_*ijkt*_	It represents the lot completion time for the product *i* to be assigned to the *kth* position onstage *j* in order *t*
*I*_*ijkk*′*t*_	It represents the difference between the total amount of inventory processed by product *i* at the *kth* position onstage *j* in order *t* and the amounts of materials that need to be processed and consumed at position *k*′ of stage *j*+1, that is, the intermediate inventory
*I*_*ij*_^max^	It represents the maximum inventory ability of product *i* at stage *j*
*λ*_*ij*_	It represents the production and processing time coefficient of product *i* at stage *j*
*q*_*ij*_	It represents the time required to adjust and clean up the equipment when product *i* switches onstage *j*
*d*_*it*_	It represents the demand for the product *i* in order *t*
*Z*	It represents outlook period
*prz*_*ij*_	It represents the unit switching cost of product *i* onstage j
*prk*_*ij*_	It represents the unit inventory cost of product *i* onstage *j*
*φ*_*j*_(*t*)	It represents device failure rate for equipment *i* in order *t*
*μ*_*jt*_	It represents the incremental factor of the failure rate for equipment *i* in order *t*
*T*_*ii*′*jt*_	It represents the adjustment time required for production from product *i* to product *i*′ onstage *j* in order *t*
*jm*	It represents the total number of equipment
*km*	It represents the total number of event points
*e*_*ijkk*′*t*_	It represents whether the intermediate inventory in order *t* is greater than 0, true is 1, and false is 0
*v*_*ijkk*′*t*_	It represents whether the intermediate inventory in order *t* is less than the maximum ability limit, true is 1, and false is 0
*A*	It represents a maximum positive number
*B*	It represents a minimal positive number

**Table 2:** Basic parameter setting table for process production in the chemical enterprise.

Stage and production	Original maximum production ability of equipment	Minimum production ability of equipment	Conversion rate	Fixed time factor	Variable time factor	Inventory limit	Unit inventory cost	Unit switching cost
1,1	18	5	1.0	4.0	0.10	16	12	10
1,2	18	5	1.0	3.0	0.15	17	12	10
1,3	18	5	1.0	5.0	0.12	16	12	10
1,4	18	5	1.0	4.5	0.13	16	12	10
2,1	20	5	0.9	3.0	0.10	20	10	9
2,2	20	5	0.9	2.5	0.10	22	10	9
2,3	20	5	0.9	2.6	0.21	21	10	9
2,4	20	5	0.9	2.8	0.15	23	10	9
3,1	25	5	0.8	6.0	0.10	—	—	8
3,2	25	5	0.8	5.5	0.11	—	—	8
3,3	25	5	0.8	6.4	0.09	—	—	8
3,4	25	5	0.8	5.0	0.12	—	—	8

**Table 3:** Table of demand for products in historical orders.

	DOP	DINP	DOA	DOTP
Order1	15	13	17	14
Order2	17	14	18	15
Order3	16	14	17	15
Order4	16	15	18	16
Order5	16	13	17	14
Order6	17	14	18	16
Order7	17	14	18	16
Order8	16	15	18	15
Order9	16	14	17	15
Order10	16	15	17	16
Order11	16	13	17	14
Order12	17	13	18	16
Order13	15	13	16	14
Order14	16	14	17	15
Order15	17	14	18	15
Order16	17	14	18	15
Order17	16	13	17	14
Order18	16	15	17	16
Order19	15	13	17	14
Order20	15	13	17	16
Order21	15	13	16	14
Order22	16	13	17	15
Order23	16	14	17	16
Order24	16	15	17	15

**Table 4:** Related parameter table of each equipment failure rate in historical orders.

	*μ*_1_	*η*_1_	*μ*_2_	*η*_2_	*μ*_3_	*η*_3_
Order1	1.81	74	1.31	91	1.46	83
Order2	1.89	76	1.39	92	1.44	82
Order3	1.68	74	1.33	91	1.47	84
Order4	1.65	75	1.33	92	1.61	82
Order5	1.78	76	1.36	93	1.46	81
Order6	1.85	82	1.37	90	1.45	82
Order7	1.89	75	1.39	92	1.57	81
Order8	1.77	79	1.33	91	1.57	82
Order9	1.79	76	1.33	93	1.59	84
Order10	1.62	75	1.4	92	1.5	83
Order11	1.74	79	1.52	91	1.6	82
Order12	1.86	76	1.56	93	1.59	80
Order13	1.85	75	1.52	93	1.57	82
Order14	1.82	80	1.33	91	1.52	84
Order15	1.62	76	1.34	92	1.42	82
Order16	1.89	79	1.32	90	1.52	80
Order17	1.72	82	1.36	91	1.64	83
Order18	1.84	77	1.38	92	1.65	80
Order19	1.89	80	1.41	92	1.6	81
Order20	1.74	77	1.5	90	1.57	83
Order21	1.72	79	1.36	93	1.63	84
Order22	1.72	78	1.48	92	1.62	82
Order23	1.85	76	1.4	91	1.56	84
Order24	1.86	75	1.42	90	1.61	82

**Table 5:** Lot schedule table for each product in the first stage (product and lot).

	1,1	1,2	2,1	2,2	3,1	3,2	4,1	4,2
Order1	7.65	13.18	6.86	11.20	9.54	14.07	7.43	12.01
Order2	7.34	16.27	6.55	12.89	9.33	15.67	7.52	13.31
Order3	7.58	14.64	6.42	13.02	9.56	14.05	7.44	13.39
Order4	7.56	14.66	6.47	14.36	9.75	15.25	7.43	14.79
Order5	7.50	14.72	6.52	11.54	9.85	13.76	7.65	11.79
Order6	7.46	16.15	6.58	12.86	9.77	15.23	7.69	14.53
Order7	7.40	16.21	6.70	12.74	9.73	15.27	7.57	14.65
Order8	7.76	14.46	6.65	14.18	9.65	15.35	7.55	13.28
Order9	7.52	14.70	6.53	12.91	9.69	13.92	7.61	13.22
Order10	7.67	14.55	6.32	14.51	9.61	14.00	7.64	14.58
Order11	7.43	14.79	6.45	11.61	9.57	14.04	7.58	11.86
Order12	7.56	16.05	6.66	11.40	9.68	15.32	7.60	14.62
Order13	7.35	13.48	6.31	11.75	9.64	12.58	7.55	11.89
Order14	7.39	14.83	6.64	12.80	9.88	13.73	7.59	13.24
Order15	7.30	16.31	6.65	12.79	9.91	15.09	7.62	13.21
Order16	7.47	16.14	6.56	12.88	9.92	15.08	7.67	13.16
Order17	7.64	14.58	6.38	11.68	9.88	13.73	7.45	11.99
Order18	7.55	14.67	6.60	14.23	9.76	13.85	7.65	14.57
Order19	7.54	13.29	6.66	11.40	9.77	13.84	7.69	11.75
Order20	7.33	13.50	6.87	11.19	9.83	13.78	7.50	14.72
Order21	7.68	13.15	6.22	11.84	9.70	12.52	7.53	11.91
Order22	7.76	14.46	6.54	11.52	9.58	14.03	7.65	13.18
Order23	7.77	14.45	6.68	12.76	9.56	14.05	7.68	14.54
Order24	7.57	14.65	6.18	14.66	9.54	14.07	6.18	14.66

**Table 6:** Lot schedule table for each product in the second stage (product and lot).

	1,1	1,2	2,1	2,2	3,1	3,2	4,1	4,2
Order1	5.43	15.40	5.00	13.06	7.06	16.55	5.12	14.32
Order2	5.36	18.25	5.05	14.39	6.97	18.03	5.10	15.73
Order3	5.30	16.92	5.08	14.36	6.93	16.68	5.08	15.75
Order4	5.27	16.95	5.11	15.72	6.94	18.06	5.09	17.13
Order5	5.25	16.97	5.12	12.94	6.92	16.69	5.03	14.41
Order6	5.32	18.29	5.09	14.35	6.91	18.09	5.00	17.22
Order7	5.28	18.33	5.10	14.34	7.04	17.96	5.01	17.21
Order8	5.34	16.88	5.12	15.71	7.14	17.86	5.05	15.78
Order9	5.40	16.82	5.18	14.26	7.07	16.54	5.06	15.77
Order10	5.53	16.69	5.16	15.67	6.99	16.62	5.13	17.09
Order11	5.49	16.73	5.15	12.91	7.02	16.59	5.07	14.37
Order12	5.41	18.20	5.12	12.94	7.17	17.83	5.08	17.14
Order13	5.55	15.28	5.11	12.95	6.95	15.27	5.05	14.39
Order14	5.54	16.68	5.09	14.35	6.91	16.70	5.06	15.77
Order15	5.45	18.16	5.12	14.32	7.03	17.97	5.09	15.74
Order16	5.34	18.27	5.13	14.31	6.76	18.24	5.11	15.72
Order17	5.37	16.85	5.12	12.94	6.88	16.73	5.07	14.37
Order18	5.35	16.87	5.09	15.74	6.86	16.75	5.05	17.17
Order19	5.27	15.56	5.08	12.98	6.93	16.68	5.03	14.41
Order20	5.33	15.50	5.05	13.01	6.86	16.75	5.05	17.17
Order21	5.24	15.59	5.10	12.96	6.85	15.37	5.08	14.36
Order22	5.20	17.02	5.14	12.92	6.95	16.66	5.12	15.71
Order23	5.17	17.05	5.12	14.32	6.91	16.70	5.05	17.17
Order24	5.00	17.22	5.00	15.84	6.95	16.66	5.00	15.84

**Table 7:** Lot schedule table for each product in the third stage (product and lot).

	1,1	1,2	2,1	2,2	3,1	3,2	4,1	4,2
Order1	18.75	0.00	16.25	0.00	21.25	0.00	17.50	0.00
Order2	21.25	0.00	17.50	0.00	22.50	0.00	18.75	0.00
Order3	20.00	0.00	17.50	0.00	21.25	0.00	18.75	0.00
Order4	20.00	0.00	18.75	0.00	22.50	0.00	20.00	0.00
Order5	20.00	0.00	16.25	0.00	21.25	0.00	17.50	0.00
Order6	21.25	0.00	17.50	0.00	22.50	0.00	20.00	0.00
Order7	21.25	0.00	17.50	0.00	22.50	0.00	20.00	0.00
Order8	20.00	0.00	18.75	0.00	22.50	0.00	18.75	0.00
Order9	20.00	0.00	17.50	0.00	21.25	0.00	18.75	0.00
Order10	20.00	0.00	18.75	0.00	21.25	0.00	20.00	0.00
Order11	20.00	0.00	16.25	0.00	21.25	0.00	17.50	0.00
Order12	21.25	0.00	16.25	0.00	22.50	0.00	20.00	0.00
Order13	18.75	0.00	16.25	0.00	20.00	0.00	17.50	0.00
Order14	20.00	0.00	17.50	0.00	21.25	0.00	18.75	0.00
Order15	21.25	0.00	17.50	0.00	22.50	0.00	18.75	0.00
Order16	21.25	0.00	17.50	0.00	22.50	0.00	18.75	0.00
Order17	20.00	0.00	16.25	0.00	21.25	0.00	17.50	0.00
Order18	20.00	0.00	18.75	0.00	21.25	0.00	20.00	0.00
Order19	18.75	0.00	16.25	0.00	21.25	0.00	17.50	0.00
Order20	18.75	0.00	16.25	0.00	21.25	0.00	20.00	0.00
Order21	18.75	0.00	16.25	0.00	20.00	0.00	17.50	0.00
Order22	20.00	0.00	16.25	0.00	21.25	0.00	18.75	0.00
Order23	20.00	0.00	17.50	0.00	21.25	0.00	20.00	0.00
Order24	20.00	0.00	18.75	0.00	21.25	0.00	18.75	0.00

**Table 8:** Table of objective function values for each scenario in three cases.

		1	2	3	4	5	6	7

Case 1	F1	59.9	60.1	60.6	92.0	63.1	—	—
	F2	859.4	804.5	730.6	686.1	686.2	—	—
		1	2	3	4	5	6	7

Case 2	F1	60.4	62.8	64.8	64.2	84.0	67.0	78.5
	F2	1041.6	1030.6	849.2	1011.2	620.6	740.0	641.5
		1	2	3	4	5	6	7

Case 3	F1	59.9	60.6	77.0	68.3	—	—	—
	F2	834.5	823.4	832.0	839.2	—	—	—

**Table 9:** Switching cost table for each scenario in three cases.

Case 1	1	2	3	4	5	6	7	Average
	516.7	539.2	398.3	442.8	320.2	—	—	443.4

Case 2	1	2	3	4	5	6	7	Average
	492.6	576.7	473.3	550.8	453.3	321.7	464.9	476.2

Case 3	1	2	3	4	5	6	7	Average
	517.8	546.7	546.7	546.7	—	—	—	539.5

**Table 10:** Inventory cost table for each scenario in three cases.

Case 1	1	2	3	4	5	6	7	Average
	342.7	265.3	332.3	243.3	366	—	—	310.0

Case 2	1	2	3	4	5	6	7	Average
	549	453.9	375.9	460.4	167.3	418.3	176.6	371.6

Case 3	1	2	3	4	5	6	7	Average
	316.7	276.7	285.3	292.5	—	—	—	292.8

**Table 11:** Demand table of each product predicted by the neural network.

	DOP	DINP	DOA	DOTP
Demand	16	14	17	15

**Table 12:** Relevant parameter table of the failure rate of each piece of equipment predicted by the neural network.

	*μ*_1_	*η*_1_	*μ*_2_	*η*_2_	*μ*_3_	*η*_3_
Relevant parameter	1.80	76	1.43	92	1.57	82

**Table 13:** Table of steady-state errors for each lot before and after MPC implementation (phase and product and lot).

	1,1,1	1,1,2	1,2,1	1,2,2	1,3,1	1,3,2	1,4,1	1,4,2
Before MPC	7.57	14.65	6.18	14.66	9.54	14.07	6.18	14.66
After MPC	7.56	14.68	5.00	14.46	8.96	14.68	6.17	14.68
Steady-state error	−0.13%	0.20%	19.09%	−1.36%	−6.08%	4.34%	−0.16%	0.14%
	2,1,1	2,1,2	2,2,1	2,2,2	2,3,1	2,3,2	2,4,1	2,4,2

Before MPC	5.00	17.22	5.00	15.84	6.95	16.66	5.00	15.84
After MPC	5.00	17.24	5.00	14.46	7.95	15.69	5.00	15.85
Steady-state error	0.00%	0.12%	0.00%	−8.71%	14.39%	−5.82%	0.00%	0.06%
	3,1,1	3,1,2	3,2,1	3,2,2	3,3,1	3,3,2	3,4,1	3,4,2

Before MPC	20.00	0.00	18.75	0.00	21.25	0.00	18.75	0.00
After MPC	20.00	0.00	17.50	0.00	21.25	0.00	18.75	0.00
Steady-state error	0.00%	0.00%	−6.67%	0.00%	0.00%	0.00%	0.00%	0.00%

## Data Availability

**The data supporting the current study are available from the corresponding author upon request.**
